# Maximizing Phenolics, γ-Aminobutyric Acid, and Antioxidant Capacity in White Corn Sprouts Through H_2_O_2_ Soaking Concentration and Germination Time Optimization

**DOI:** 10.3390/biology15131082

**Published:** 2026-07-06

**Authors:** Liliana León-López, Carlos Daniel Martínez-Camacho, Saraid Mora-Rochin, Luis Martín Sánchez-Magaña, Fabiola Araceli Guzmán-Ortiz, Israel Benítez-García, Edith Oliva Cuevas-Rodríguez, Cuauhtémoc Reyes-Moreno

**Affiliations:** 1 Programa de Posgrado Integral en Biotecnología, Facultad de Ciencias Químico Biológicas, Universidad Autónoma de Sinaloa, Ciudad Universitaria, Culiacán 80000, Sinaloa, Mexico; calemartinez98@gmail.com (C.D.M.-C.); luis_magana@uas.edu.mx (L.M.S.-M.); edith.cuevas.r@uas.edu.mx (E.O.C.-R.); creyes@uas.edu.mx (C.R.-M.); 2 Posgrado en Ciencia y Tecnología de Alimentos, Facultad de Ciencias Químico Biológicas, Universidad Autónoma de Sinaloa, Ciudad Universitaria, Culiacán 80000, Sinaloa, Mexico; 3 SECIHTI-Área Académica de Química, Universidad Autónoma del Estado De Hidalgo, Ciudad del Conocimiento, Mineral de la Reforma 42184, Hidalgo, Mexico; fabiguzman01@yahoo.com.mx; 4 Programa de Maestría en Ciencias Aplicadas, Unidad Académica de Ingeniería en Biotecnología, Universidad Politécnica de Sinaloa (UPSIN), Mazatlán 82199, Sinaloa, Mexico; ibenitez@upsin.edu.mx

**Keywords:** germination, H_2_O_2_-elicitation, white corn sprouts

## Abstract

White corn (*Zea mays* L.) is a staple grain in the Mexican diet and an important source of carbohydrates, proteins, dietary fiber, vitamins, minerals, and bioactive compounds. This study evaluated the effects of H_2_O_2_ elicitation and germination time on germination performance, bioactive compounds, and antioxidant capacity of white corn sprouts. Response surface methodology, based on a rotatable central composite design (13 treatments) was used to optimize H_2_O_2_ concentration (0–50 mM) during 24 h soaking and germination times ranging from 0 to 96 h. Optimal conditions were 20 mM H_2_O_2_ and 92 h germination, yielding 94% germination, 236.94 mg GAE/100 g free phenolic content, 21.63 mg/100 g GABA, and 6311.06 µmol TE/100 g antioxidant capacity (ABTS). Under these conditions, protein, GABA, and gallic acid contents, as well as antioxidant activity, increased by 9.26%, 34.01%, 30.95%, and 27.24%, respectively, compared with germinated grains without elicitor treatment. These findings demonstrate that H_2_O_2_-assisted germination is an effective strategy for enhancing the nutritional and functional properties of white corn sprouts.

## Simple Summary

To strengthen food sovereignty in Mexico, this study highlights H_2_O_2_-assisted sprouting as a low-cost strategy for enhancing the nutritional value of white corn. Soaking kernels for 24 h followed by a 92 h germination period significantly increased the protein content and antioxidant bioactive compounds. This practical bioprocess offers a sustainable, accessible alternative to improve the health benefits of a national staple, providing an effective tool to combat malnutrition and enhance dietary quality across society.

## 1. Introduction

Corn is Mexico’s most important crop and one of the world’s most significant crops because of its food, political, economic, social, and cultural significance [1]. It is a staple ingredient and major energy source in the Mexican diet. Corn (*Zea mays* L.) is a rich source of carbohydrates, proteins, dietary fiber, vitamins, minerals, and bioactive compounds with antioxidant properties that may help protect against free radical-induced degenerative diseases [2]. Although corn is consumed in diverse forms depending on locality or ethnicity [3], processing methods can significantly affect the content and bioavailability of its bioactive compounds [4,5]. In Mexico, corn is primarily consumed as tortillas, the main vehicle for nutrient intake. Traditionally, it is processed through nixtamalization, or alkaline cooking, a process that, while fundamental to traditional diets, causes the leaching of critical components (such as proteins, soluble carbohydrates, dietary fiber, vitamins, and polyphenols) into the wastewater by-product known as nejayote, which creates a heavy environmental burden [2,5,6]. Given this context, it is imperative to investigate and implement cleaner technological alternatives, such as extrusion cooking or controlled sprouting, which can mitigate environmental impacts by reducing resource consumption while simultaneously enhancing nutritional density and bioaccessibility of bioactive compounds within the processed grain [4,6]. Germination is an effective bioprocess for enhancing bioactive compounds levels in corn grains. It begins with water imbibition and ends with radicle emergence through the seed coat, triggering transcription, translation, cell division, and metabolic activity. These changes increase γ-aminobutyric acid (GABA), and phenolic compounds, as well as antioxidant activity, compared with non-germinated grains [2,7]. Recent studies have shown that pre-germination stress induced by elicitors increases bioactive compounds in sprouted grains, and that their effects vary with germination conditions [8,9].

Elicitors can be classified into two main categories: biotic and abiotic. Biotic elicitors are derived from living organisms or their structural components and trigger plant defense responses against pathogens and adverse environmental conditions. These include chitosan, oligosaccharides, phytohormones, bacterial metabolites, beneficial microorganisms, and various phytochemicals [10,11]. Abiotic elicitors include physical treatments such as ultraviolet radiation, ultrasound, thermal stress, and pulsed electric fields, as well as chemical agents including metal ions, salicylic acid, jasmonic acid, and hydrogen peroxide (H_2_O_2_) [10]. Among these, H_2_O_2_ has attracted considerable attention because, although traditionally regarded as a toxic reactive oxygen species (ROS), it also functions as an important signaling molecule involved in the regulation of seed germination and early seedling development [12]. H_2_O_2_ has been reported to act as a secondary messenger that modulates redox homeostasis and interacts with plant hormones, particularly abscisic acid (ABA) and gibberellins (GA), thereby promoting dormancy release, reserve mobilization, cell wall loosening, and radicle protrusion [12,13]. Furthermore, H_2_O_2_ activates antioxidant defense systems and stress-responsive metabolic pathways, leading to the accumulation of phenolic compounds, flavonoids, γ-aminobutyric acid (GABA), and other bioactive metabolites, thereby enhancing nutritional and functional properties [12,14]. Previous studies have reported increased glucosinolate accumulation and antioxidant capacity in broccoli sprouts [15], improved germination performance and antioxidant metabolism in pea seedlings through the regulation of endogenous signaling networks [14], and enhanced phenolic and flavonoid contents in amaranth sprouts treated with 340 ppm H_2_O_2_ [16]. These findings suggest that H_2_O_2_-mediated elicitation can stimulate the biosynthesis of health-promoting compounds during germination, although its effects may vary with species and germination conditions.

Response surface methodology (RSM), using a central composite rotatable design (CCRD) with axial points beyond factorial extremes, efficiently optimizes such processes. CCRD enables precise response prediction, nonlinear modeling, curvature detection, robust multiparameter optimization, and quadratic polynomial fitting with fewer runs [17,18]. Based on the reported role of H_2_O_2_ as a signaling molecule involved in the activation of stress-responsive metabolic pathways during germination, we hypothesized that H_2_O_2_ elicitation and germination time interact synergistically to enhance the accumulation of phenolic compounds and γ-aminobutyric acid (GABA), thereby increasing the antioxidant capacity and nutraceutical value of white corn sprouts. Therefore, this study aimed to evaluate the effects of H_2_O_2_ concentration and germination time on the nutraceutical properties of white corn sprouts and, using response surface methodology, to identify the optimal conditions for maximizing total phenolic content, GABA concentration, and antioxidant activity.

## 2. Materials and Methods

### 2.1. Materials

White corn (*Zea mays* L.) grains (commercial hybrid Hipopótamo) used in this study were purchased from a local market in Culiacán, Sinaloa, Mexico, in 2022. The seeds were cleaned and stored in plastic bags at 4 °C until subsequent germination tests and chemical analyses.

### 2.2. White Corn Germination Process

The germination protocol was modified from León-López, Escobar-Zúñiga, Salazar-Salas, Mora-Rochín, Cuevas-Rodríguez, Reyes-Moreno and Milán-Carrillo [8]. White corn grains were surface-sterilized by immersion in 0.5% sodium hypochlorite (1:5 *w*/*v* ratio) for 10 min, the solution was then discarded, and seeds were rinsed three times with purified water. Batches of 20 g of seeds were then soaked (seed: water ratio, 1:5 *w*/*v*) for 24 h at room temperature in varying H_2_O_2_ concentrations (0–50 mM) (Table 1). After soaking, the seeds were rinsed with distilled water and uniformly distributed on filter paper folds within plastic containers. Seed were germinated in an incubator chamber (I-36VL Model, Percival Scientific Inc., Perry, IA, USA) at 25 °C and 80–90% relative humidity until the times specified in the experimental design (0–96 h) (Table 1).

### 2.3. Experimental Design

The independent variables were hydrogen peroxide soaking concentration: (X_1_ = [H_2_O_2_], 0–50 mM) and germination time (X_2_ = Gt, 0–96 h). A central composite rotatable design (CCRD) with 13 runs (Table 1) was used. The runs were randomized before execution to minimize potential systematic bias. Four independent lots were produced for each of the experimental conditions. Two lots were used to assess germination percentage, sprout length, diameter, and the number of secondary roots. The remaining two lots were dried at 55 °C, milled to pass through a US No. 80 sieve, and stored at −20 °C until the determination of free phenolic content, GABA concentration, and antioxidant activity. All analytical measurements were conducted in triplicate.

### 2.4. Germination Percentage

The germination percentage (GP) was assessed for seeds treated with varying concentrations of H_2_O_2_ concentrations and germination times (Table 1). GP was calculated as the number of germinated seeds (radicle > 1 mm) divided by the total number of seeds sown per treatment, multiplied by 100 [8].

### 2.5. Sprout Growth

At the end of each treatment, the physical characteristics of the obtained corn sprouts were evaluated. Radicle length and diameter were recorded using a digital caliper (Truper CALDI-6MP), and the number of secondary roots was counted.

### 2.6. Flour Sample Obtaining Process

Sprouts from each treatment were dried at 55 °C for 24 h in a food dehydrator (Hamilton Beach, 32100A, 500 W, Glen Allen, VA, USA), then ground using a coffee grinder (Hamilton Beach, 80350R, Glen Allen, VA, USA). The resulting flours were sifted through an 80-mesh sieve (0.180 mm aperture) and stored at 4 °C until analysis.

### 2.7. Preparation of Phytochemical Extracts

#### 2.7.1. Free Extract

Free phytochemical extracts were obtained following the methodology described by Adom and Liu [19] with some modifications. Flour samples (0.05 g) were suspended in 1 mL 80% ethanol (*v*/*v*), agitated for 30 min in a horizontal rotary shaker (RKVSD, ART Inc. Laurel, MD, USA) (200 rpm, 25 °C), and centrifuged (Eppendorf 5810R, AG, Hamburg, Germany) at 5000× *g*, 4 °C for 10 min. The supernatants were collected, evaporated to dryness, and resuspended in either 200 μL 50% methanol (for phenolic content and antioxidant capacity assays) or 70% ethanol (for GABA quantification). The extracts were stored at −20 °C until analysis and prepared in triplicate.

#### 2.7.2. Bound Extract

Bound phytochemical extracts were obtained following the methodology described by Adom and Liu [19], as modified by Mora-Rochin, et al. [20]. The insoluble residue from free phytochemical extraction was digested with 1 mL 2 M NaOH and subjected to thermal treatment (30 min, 90 °C water bath). The sample was then agitated for 1 h at room temperature, acidified with 200 μL concentrated HCl, and defatted with 500 μL hexane. The mixture was extracted four times with 500 μL ethyl acetate. The ethyl acetate fraction was collected, evaporated to dryness (Speed Vac Concentrator, Thermo Electron Corporation, Waltham, MA, USA), reconstituted in 200 μL 50% methanol, and stored at −20 °C until analysis. The extractions were performed in triplicate.

### 2.8. Phytochemical Determination

#### 2.8.1. Phenolic Content

The phenolic content was quantified using the Folin–Ciocalteu colorimetric method [21]. Briefly, 20 µL of diluted extracts (obtained as in Section 2.7.1 and Section 2.7.2) were mixed with 180 µL of Folin–Ciocalteu reagent and incubated for 20 min. The absorbance of the resulting blue complex was measured at 750 nm using a Synergy HT Multi-Detection microplate reader (BioTek Instruments, Inc., Winooski, VT, USA). The free phenolic content (FPC) values were determined from a gallic acid calibration curve (0–300 mg/L) and expressed as mg gallic acid equivalents (GAE) per 100 g dry weight (DW) of flour (mg GAE/100 g DW). The content determined in the free extract was designated as FPC, while that determined in the bound extract was designated as bound phenolic content (BPC). The total phenolic content (TPC) was calculated by summing the FPC and BPC.

#### 2.8.2. γ-Aminobutyric Acid Content

The GABA content in the germinated corn flour was determined using the method reported by Watchararparpaiboon, et al. [22]. An aliquot (0.1 mL) of extract (obtained as described in Section 2.7.1) was mixed with 0.2 mL borate buffer (0.2 M boric acid/sodium borate, pH 9) and 1 mL 6% phenol reagent, homogenized, and cooled on ice. After adding 0.4 mL 7.5% sodium hypochlorite, the mixture was shaken on ice and boiled for 10 min, and then cooled in ice water for 5 min. The absorbance was measured at 630 nm against a reagent blank. Calibration used GABA (Sigma, St. Louis, MI, USA) standard (0–600 µg/mL) prepared from a 500 mg/mL stock in deionized water. The results were expressed as mg GABA/100 g DW.

### 2.9. Antioxidant Capacity

#### ABTS Assay

The antioxidant capacity (AOxC) of sprouted seed flours was assessed using the ABTS assay [23]. The sample extract (7.5 μL) was added to a 96-well plate and diluted to 300 μL with ABTS•^+^ (2,2′-azinoazino-bis (3-ethylbenzothiazoline-6-sulfonic acid) solution (final volume per well). After 10 min incubation at room temperature, absorbance was measured at 735 nm (Synergy HT Multi-Detection microplate reader; BioTek Instruments, Inc., Winooski, VT, USA). Trolox (0–800 µg/mL) was used as the standard, and the results were expressed as µmol Trolox equivalents (TE) per 100 g dry weight (µmol TE/100 g DW).

### 2.10. Regression Analysis and Optimization

The optimal H_2_O_2_ soaking concentration ([H_2_O_2_]) and germination time (Gt) to maximize the germination percentage, free phenolic content (FPC), GABA, and antioxidant capacity (AOxC) in the ABTS assay of sprouted white corn were determined using response surface methodology (RSM). The central composite rotatable design (CCRD) included 13 randomized runs (Table 1), with [H_2_O_2_] varying from 0 to 50 mM and Gt from 0 to 96 h. FPC, GABA, and AOxC were modeled as quadratic responses according to Equation (1):

(1) $$Y=\beta_{0}+\sum_{i=1}^{2}\beta_{i}X_{i}+\sum_{i=2}^{2}\beta_{ii}X_{i}^{2}+\sum_{i=1}^{2}\sum_{i=j+1}^{2}\beta_{ij}X_{i}X_{j}+\epsilon$$

where Y is the predicted response variable (Y_1_ = PG, Y_2_ = FPC, Y_3_ = GABA, Y_4_ = AOxC); β_0_, β_i_, β_ii_, and β_ij_ represent the intercept, linear, quadratic, and interaction regression coefficients, respectively; X_i_ and X_j_ are the independent variables (X_1_ = [H_2_O_2_], X_2_ = Gt); and ε is the experimental error. Non-significant terms (*p* > 0.10) were removed from the full second-order polynomial model using a backward stepwise regression. The reduced model was subsequently used to predict each response variable and to generate response surfaces [24]. Model adequacy was assessed using analysis of variance (ANOVA), coefficient of determination (R^2^), adjusted R^2^, predicted R^2^, and lack-of-fit tests. All statistical analyses, model fitting, and optimization procedures were performed using Design-Expert^®^ software version 13 (Stat-Ease Inc., Minneapolis, MN, USA) [25].

### 2.11. Optimal Sprouts Characterization

#### 2.11.1. Proximate Chemical Composition

The moisture, protein, lipid, and ash contents of white corn and its sprouted counterparts, under both optimized and control conditions, were quantified using the official AOAC International methods [26]. The carbohydrate content was determined by difference, subtracting the combined weights of moisture, protein, lipid, and ash from 100 g of the sample. All values were reported as g/100 g on a dry weight (DW) basis.

#### 2.11.2. Antioxidant Capacity

##### ORAC Assay

Additionally, the antioxidant capacity (AOxC) of unprocessed white corn and sprouted grains produced under optimized and control conditions ([H_2_O_2_] = 0 mM, Gt = 92 h) was measured using the oxygen radical absorbance capacity (ORAC) assay [27]. Peroxyl radicals were generated using AAPH (2,2′-azobis(2-amidinopropane) dihydrochloride), and fluorescence decay was quantified in a microplate reader (Synergy HT Multi-Detection; BioTek Instruments, Inc., Winooski, VT, USA). For each reaction, 150 µL of fluorescein (0.1 µM) was dispensed, mixed, and incubated for 30 min before adding 25 µL of AAPH. The reaction was carried out at 37 °C, and the fluorescence (485 nm for excitation and 538 nm for emission) was measured at 2 min intervals for 60 min. Results were expressed as µmol Trolox equivalents (TE) per 100 g dry weight (DW) basis (µmol TE/100 g DW).

#### 2.11.3. Identification and Quantification of Free and Bound Phenolic Compounds by HPLC

The phenolic profiles of the free and bound extracts were analyzed using a Dionex UltiMate 3000 HPLC system equipped with a photodiode array detector (DAD3000; Thermo Fisher Scientific, New York, NY, USA). An injection volume of 10 µL was used. Separation was performed on a C18 Acclaim 120 Å analytical column (5 μm, 120 Å, 4.6 × 250 mm; Dionex, Thermo Fisher Scientific, New York, NY, USA) at room temperature, employing gradient elution with acetic acid–acidified water (pH 2.8; solvent A) and acetonitrile (solvent B). The 45 min gradient program was as follows: 95% A (0–8 min); 6–12% B (8–14 min); 12–20% B (14–18 min); 20–35% B (18–24 min); 35–95% B (24–27 min); 95–60% B (27–31 min); 60–40% B (31–34 min); 40–20% B (34–38 min); 20–5% B (38–45 min), at a flow rate of 0.5 mL/min, and the temperature of the column was set at 25 °C. The detection wavelengths were 280, 320, and 360 nm. Peaks were identified by matching retention times and UV-Vis spectra to authentic standards. Samples were injected in triplicate, with data processed using Chromeleon 7.0.200 software (Thermo Fisher Scientific, Sunnyvale, CA, USA) [28]. The results were reported as µg/g flour sample on a dry weight (DW) basis.

### 2.12. Statistical Analysis

The effects of H_2_O_2_ treatment on seedling growth, chemical composition, FPC, GABA, AOxC, and phenolic profile were evaluated using one-way analysis of variance (ANOVA). Mean separations were performed with Tukey’s test at the 95% confidence level (*p* < 0.05).

## 3. Results

### 3.1. Influence of H_2_O_2_ Soaking and Germination Time on Growth Performance of White Corn Sprouts

**Figure 1:**
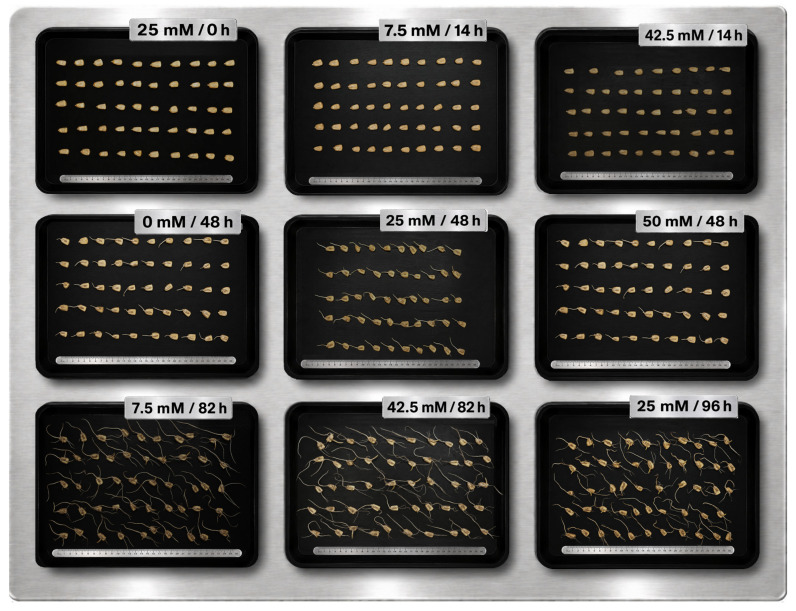
Visual appearance of white corn sprouts subjected to different H_2_O_2_ soaking concentrations (0–50 mM) and germination times (0–96 h).

The evaluated growth parameters of the sprouts included radicle length and diameter, as well as the number of secondary roots across the 13 treatments generated by the experimental design (Table 1) as shown in Table 2. When analyzing data from treatments with the same germination time (Gt), a trend toward greater root length was observed in treatments with higher [H_2_O_2_]. For treatments with Gt = 48 h, root length increased with increasing [H_2_O_2_]. The treatment with [H_2_O_2_] = 0 mM produced the shortest radicles (21.5 mm), whereas [H_2_O_2_] = 50 mM produced the longest (30.06 mm). This trend was also evident in Gt = 82 h treatments, where H_2_O_2_ concentrations ranged from 7.5 mM to 42.5 mM, resulting in nearly a 10 mm difference in average radicle length between treatments. Additionally, within the same [H_2_O_2_], radicle length increased with Gt. This was particularly clear in the [H_2_O_2_] = 25 mM treatment, suggesting that germination time exert a greater influence on root growth that [H_2_O_2_]. Conversely, H_2_O_2_ had no substantial effect on radicle diameter (0.14–0.93 mm), which increased primarily with Gt regardless of H_2_O_2_ concentration. This trend is clear when comparing Gt = 14 h treatments with those at 48, 82, and 96 h. Similarly, secondary root number showed minimal response to H_2_O_2_ concentration within the same Gt but increased markedly with longer Gt (Table 2, Figure 1).

### 3.2. Predictive Models for Germination Percentage (GP), Free Phenolic Content (FPC), γ-Aminobutyric Acid (GABA), and Antioxidant Capacity (AOxC)

**Figure 2:**
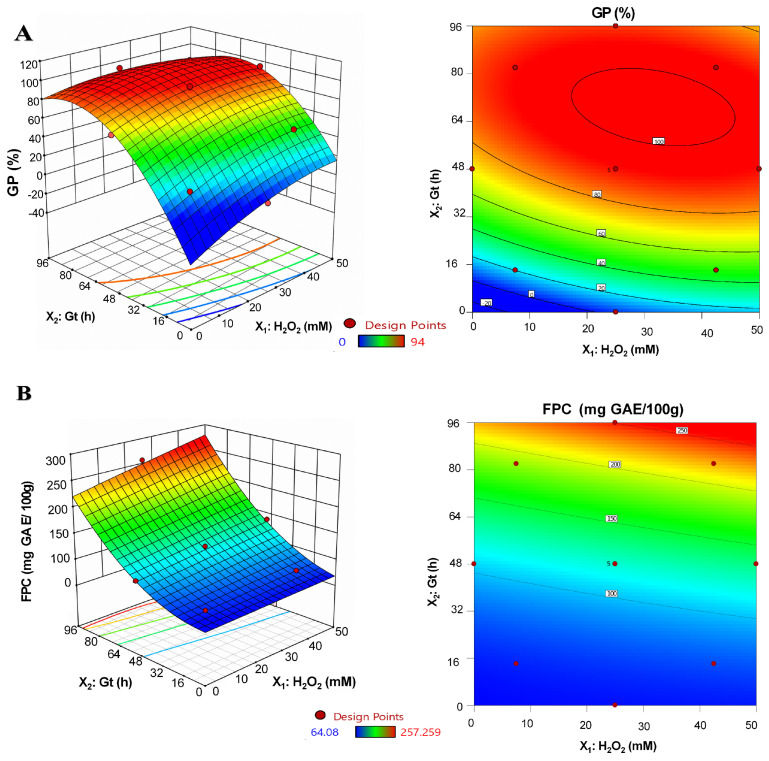
Response surface and contour plots showing the effects of soaking peroxide concentration (H_2_O_2_, mM) and germination time (Gt, h) on the response variables: (**A**) germination percentage (GP), (**B**) free phenolic compounds (FPC). The color gradient in the response surface and contour plots represents the predicted values of the response variable estimated by the fitted polynomial model. The red dots in the graphs represent the experimental design points (experimental runs) used to fit the response surface models.

**Figure 3:**
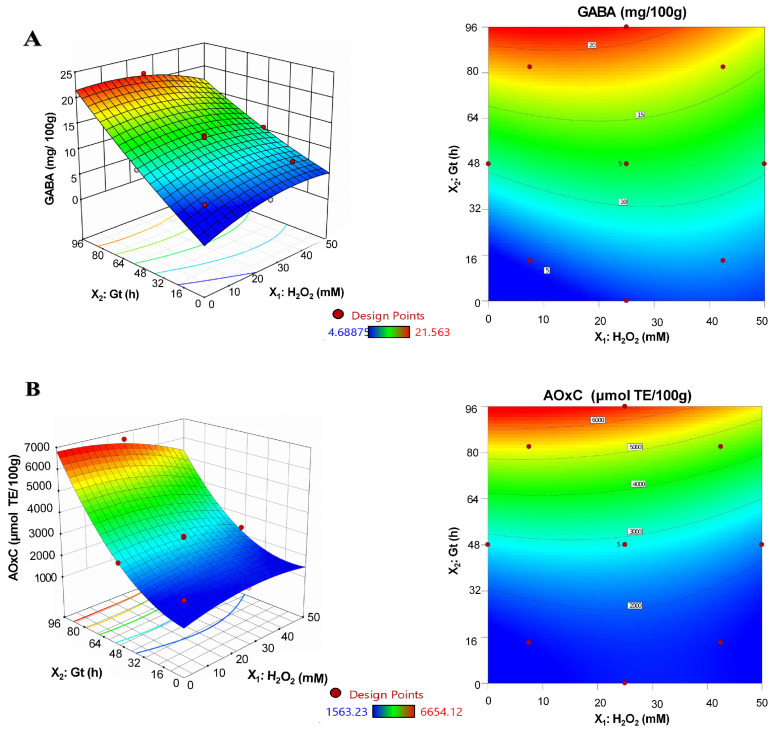
Response surface and contour plots showing the effects of soaking peroxide concentration (H_2_O_2_, mM) and germination time (Gt, h) on the response variables: (**A**) γ-aminobutyric acid (GABA), and (**B**) AOxC, antioxidant capacity by the ABTS assay of white corn sprouts. The color gradient in the response surface and contour plots represents the predicted values of the response variable estimated by the fitted polynomial model. The red dots in the graphs represent the experimental design points (experimental runs) used to fit the response surface models.

GP, FPC, GABA, and AOxC were significantly influenced by the process variables: [H_2_O_2_] and Gt, as shown in Table 3. Multiple regression analysis yielded quadratic polynomial equations that adequately described the experimental germination data for each response variable. Response surfaces and contour plots, derived from these equations, were generated to visualize the effects of [H_2_O_2_] and (Gt) (Figure 2 and Figure 3).

#### 3.2.1. Germination Percentage

GP in white corn grains was strongly affected by the process variables [H_2_O_2_] and Gt, with values ranging from 24% to 94% across the 13 evaluated treatments, depending on the experimental conditions applied (Table 3). Analysis of variance revealed a significant quadratic model for GP (*p* < 0.0001) (Table 4), which included the linear effects of [H_2_O_2_] and Gt, quadratic effects of [H_2_O_2_] and Gt, and the interaction effect between [H_2_O_2_] and Gt ([H_2_O_2_] × Gt) (Equations (2) and (3). GP increased with increasing H_2_O_2_ concentration and germination time.

Equation with coded factors,

(2) $$Y_{GP}=92.34+7.67X_{1}+30.39X_{2}-6.31X_{1}X_{2}-5.15X_{1}^{2}-22.90X_{2}^{2}$$

Equation with uncoded factors,

(3) $$GP=-30.22487+1.7635{[H}_{2}O_{2}]+3.0667Gt-0.010515{[H}_{2}O_{2}]*Gt-0.016492{{[H}_{2}O_{2}]}^{2}-0.019878{Gt}^{2\;\;}$$

#### 3.2.2. Free Phenolic Content

The free phenolic content (FPC) of germinated white corn grains was affected by the process variables [H_2_O_2_] and Gt, showing values ranging from 64.08 to 257.26 mg GAE/100 g DW (Table 3). Analysis of variance revealed a significant quadratic model for FPC (*p* < 0.0001) (Table 4), which includes the linear terms [H_2_O_2_] and Gt, the quadratic terms of Gt ([H_2_O_2_]^2^ and Gt^2^), and the interaction effect between [H_2_O_2_] and Gt ([H_2_O_2_] × Gt) (Equations (4) and (5)). Increasing H_2_O_2_ concentration and germination time positively affected the total phenolic compounds concentration.

Equation with coded factors,

(4) $$Y_{FPC}=119.92+10.72X_{1}+65.06X_{2}-6.80X_{1}X_{2}+19.36X_{2}^{2}$$

Equation with uncoded factors,

(5) $$FPC=65.07495+0.062098{[H}_{2}O_{2}]+0.020168Gt+0.011399{[H}_{2}O_{2}]*Gt+0.016804\;{Gt}^{2}$$

The contour plot in Figure 2B shows that both [H_2_O_2_] and Gt increase FPC; however, Gt has a stronger effect on FPC accumulation, with longer Gt yielding higher FPC levels. The stress produced by exposure to higher H_2_O_2_ concentrations (25, 42.5, and 50 mM) induced greater FPC accumulation in sprouts than that observed at lower H_2_O_2_ concentrations (0 and 7.5 mM), which was also observed when comparing treatments with the same Gt but different H_2_O_2_ concentrations (Table 3).

#### 3.2.3. γ-Aminobutyric Acid (GABA)

The GABA content in germinated white corn grains was affected by the process variables, ranging from 4.69 to 21.56 mg/100 g DW (Table 3). The analysis of variance produced a significant quadratic model for GABA (*p* < 0.0001) (Table 4), which included the linear terms for [H_2_O_2_] and Gt, the quadratic effects of [H_2_O_2_] and Gt ([H_2_O_2_]^2^ and Gt^2^), and their interaction effect ([H_2_O_2_] × Gt) (Equations (6) and (7)). GABA accumulation was favored by extended germination times and low-to-moderate H_2_O_2_ concentrations. Among the evaluated factors, germination time had the greatest influence, with substantially lower GABA content observed during shorter germination periods. The results indicate that the stimulatory effect of H_2_O_2_ on GABA biosynthesis became more pronounced as germination progressed, suggesting a significant interaction between elicitor concentration and germination time.

Equation with coded factors,

(6) $$Y_{GABA}=12.35+0.59X_{1}+5.63X_{2}-1.03X_{1}X_{2}-0.915X_{1}^{2}+0.43X_{2}^{2\;\;}$$

Equation with uncoded factors,

(7) $$\begin{array}{cc} GABA=1.44413 & +0.22568{[H}_{2}O_{2}]+0.17305Gt-1.72357E-003{[H}_{2}O_{2}]\;*\;Gt-2.92614E-003{{[H}_{2}O_{2}]}^{2}\\ & +3.728232{E-004Gt}^{2} \end{array}$$

In Figure 3A, the contour plot illustrates the interaction between [H_2_O_2_] and Gt on GABA accumulation. The response surface indicated that Gt exerted a greater effect than [H_2_O_2_], and extended germination times were associated with significantly higher GABA levels. Conversely, a significant interaction between [H_2_O_2_] and Gt was observed. Within the 7.5–25 mM range, increasing [H_2_O_2_] accelerated GABA accumulation, reaching a maximum at [H_2_O_2_] = 25 mM and Gt = 96 h (21.56 mg GABA/100 g, DW). At higher concentrations (42.5–50 mM), GABA content declined as Gt increased, indicating that elevated [H_2_O_2_] interacts with Gt to inhibit GABA accumulation. This pattern was also evident when comparing treatments at Gt = 82 h with [H_2_O_2_] = 7.5 and 42.5 mM, which yielded 18.44 and 16.11 mg GABA/100 g, respectively (Table 3).

#### 3.2.4. Antioxidant Capacity (AOxC)

The AOxC of germinated corn grains was affected by the process variables [H_2_O_2_] and Gt, with values ranging from 1563.23 to 6654.12 µmol TE/100 g DW (Table 3). Analysis of variance revealed a significant quadratic model for AOxC (*p* < 0.0001) (Table 4), which included the linear terms of [H_2_O_2_] and Gt, the quadratic terms ([H_2_O_2_]^2^ and Gt^2^), and their interaction effect ([H_2_O_2_] × Gt) (Equations (8) and (9). Antioxidant capacity (AOxC) increased with increasing germination time under low-to-moderate H_2_O_2_ concentrations. Nevertheless, at higher H_2_O_2_ levels, AOxC decreased despite prolonged germination, indicating that excessive H_2_O_2_ reduced antioxidant accumulation under these experimental conditions.

Equation with coded factors,

(8) $$Y_{AOxC}=2854.02-195.86X_{1}+1690.71X_{2}-221.91X_{1}X_{2}-181.90X_{1}^{2}-611.62X_{2}^{2}$$

Equation with uncoded factors,

(9) $$AOxC=1155.60575+35.77730{[H}_{2}O_{2}]+8.09055Gt-0.36985{[H}_{2}O_{2}]\;*\;Gt-0.58208{{[H}_{2}O_{2}]}^{2}-0.53092{Gt}^{2}$$

Figure 3B presents the contour plot for AOxC, illustrating the interaction effect between the process variables [H_2_O_2_] and Gt on this response variable. The results indicate that Gt exerted a greater influence than [H_2_O_2_] on AOxC accumulation, with AOxC values increasing as germination time progressed. The highest experimental value (6654.12 µmol TE/100 g DW) was obtained in the treatment with [H_2_O_2_] = 25 mM and Gt = 96 h (Table 3).

### 3.3. Optimization of H_2_O_2_ Soaking Concentration and Gt

**Figure 4:**
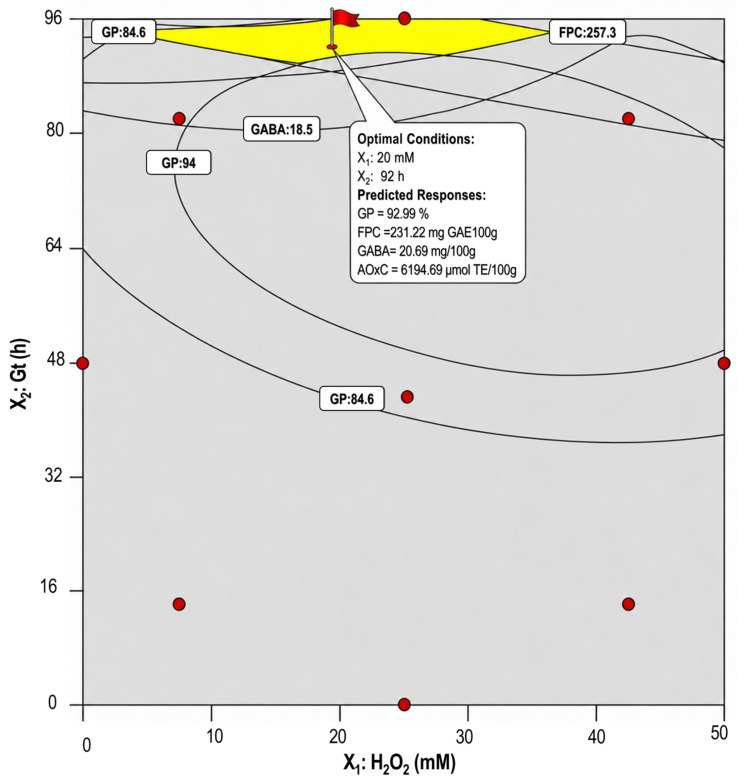
Optimization graph for the response variables: GP; Germination percentage FPC; Free phenolic content, GABA; γ-aminobutyric acid, AOxC; antioxidant capacity by the ABTS assay of white corn sprouts. [H_2_O_2_]; soaking hydrogen peroxide concentration (mM): Gt; Germination time (h). The red dots in the graph represent the experimental design points (experimental runs) used to fit the response surface models.

To optimize the process conditions, response surface methodology (RSM) was applied using a graphical approach to determine the optimal combination of [H_2_O_2_] and Gt that maximized GP, FPC, GABA, and AOxC values in the sprouts. The overlaid contour plot (Figure 4) was used to identify the optimal process conditions. The conditions [H_2_O_2_] = 20 mM and Gt = 92 h were selected as the optimal treatment based on process practicality and safety considerations, since longer germination times showed a greater tendency toward contamination. The predicted values at the optimal point under the aforementioned conditions were: GP = 92.99%, FPC = 231.26 mg GAE/100 g, DW, GABA = 20.69 mg/100 g, DW, and AOxC = 6195.87 µmol TE/100 g, DW. To validate the efficiency of the prediction model, white corn grains were germinated using the optimal process conditions obtained. Table 5 presents the values predicted by the mathematical models, their corresponding 95% confidence intervals, and the experimental results obtained for the response variables from three independent replicates produced under the optimized conditions. Comparison of the experimental and predicted values revealed that all observed responses fell within the respective confidence intervals, demonstrating good agreement between model predictions and experimental outcomes. These findings indicate that the optimized conditions were reliable and reproducible and confirm the adequacy of the developed models for accurately predicting the measured responses. Therefore, the results support the validity and practical applicability of the established optimization conditions.

#### 3.3.1. Effect of the Optimal Treatment on Growth Performance of White Corn Sprouts

Root length, mesocotyl/coleoptile length, and number of secondary roots were analyzed to assess the effect of H_2_O_2_ soaking on the growth of white corn sprouts. The treatments compared included the optimal treatment ([H_2_O_2_] = 20 mM, Gt = 92 h) and the germinated control ([H_2_O_2_] = 0, Gt = 92 h). The optimal treatment promoted greater root and mesocotyl/coleoptile (*p* ≥ 0.05) (Table 6). Relative to the control sprouts, the optimal treatment increased root and mesocotyl/coleoptile lengths by 15.76% and 28.99%, respectively.

#### 3.3.2. Effect of the Optimal Treatment on Proximate Chemical Composition of White Corn Sprouts

The proximate composition of ungerminated white corn grains was consistent with values previously reported in the literature: protein (6.4–11.4 g/100 g), lipids (3.1–7.1 g/100 g), ashes (1.1–1.5 g/100 g), and carbohydrates (83.7–86.9 g/100 g) [29,30] (Table 6). Germination led to a significant increase (*p* ≤ 0.05) in protein content; however, no significant differences were detected between the optimal treatment and the control. Both germinated treatments exhibited lower lipid contents than ungerminated seeds. Significant differences (*p* ≤ 0.05) were observed between the germinated treatments, with the optimal treatment showing a lower lipid content than the germinated control.

#### 3.3.3. Effect of the Optimal Treatment on Phytochemicals, and Antioxidant Properties of White Corn Sprouts

In unsprouted and sprouted corn, total phenolic content in the free fraction ranged from 27.29 to 95.45 µg/g DW, whereas the bound fraction showed values from 54.15 to 79.34 µg/g DW, respectively. Unsprouted corn exhibited the lowest free fraction of total phenolic compounds (27.29 µg/g DW; *p* < 0.05), while its bound fraction was the highest (79.34 µg/g DW; *p* < 0.05). Baranzelli, et al. [31] analyzed the free and bound phenolic fractions in wheat; these authors observed that after germination for 72 h, the free phenolic fraction increased by 58% (*p* < 0.05).

The optimal germination treatment significantly increased GABA content from 5.84 to 21.63 mg/100 g DW (+261.94%), and produced 34.01% increase compared with the germinated control (16.14 mg/100 g DW; Table 6). Hiran, et al. [32] reported 5.84 mg/100 g GABA in ungerminated white corn, which is consistent with our ungerminated seed value; they also reported 15.7 mg/100 g GABA in corn sprouts at 72 h, which is similar to the control treatment.

Regarding antioxidant capacity assessed by the ABTS method, both the optimal and control germination treatments showed significant increases (*p* < 0.05) compared with ungerminated seeds in the free (+416.83% and +379.40%), bound (+41.48% and +29.55%), and total (+116.33% and +99.32%) extracts. Furthermore, the optimal treatment exhibited significantly higher antioxidant capacity than the control treatment in the free (+7.81%), bound (+9.21%), and total (+8.53%) extracts (*p* < 0.05).

Results obtained by the ORAC method showed the same trend as those obtained by the ABTS method (Table 6), with a significant increase (*p* < 0.05) in the optimal and control treatments compared with the ungerminated grains in the free (+139.24% and +88.90%), bound (+63.88% and +22.30%), and total (+87.59% and +43.25%) phenolic fractions. Likewise, the optimal germination treatment exhibited significantly higher values (*p* < 0.05) than the control germination treatment in the free (+26.65%), bound (+34.00%), and total phenolic fractions (+30.95%).

It has been reported that the H_2_O_2_ stress treatment applied during soaking induced specific alterations in the free and bound phenolic fractions compared with those of both unprocessed seeds and control sprouts [9]. Regarding the phenolic compound profile of the samples, ferulic acid was the predominant phenolic acid in unsprouted corn, with the bound fraction accounting for 77% of the total phenolic content. This study shows that sprouting significantly alters total phenolic acid content.

Specifically, the bound fraction decreased by more than 28% (*p* < 0.05) after germination, whereas the free fraction increased significantly (*p* < 0.05) relative to unsprouted corn (27.29 µg/g DW), reaching 91.38 µg/g DW in the optimized treatment and 95.45 µg/g DW in the control. All analyzed phenolic acids and the flavonoid responded to this increase, showing 1.1—to 5.5-fold higher levels in the free fraction, while in the bound fraction increases of 1.06—to 2.44-fold were observed under control and optimal sprouting conditions, respectively, compared with unsprouted corn. Conversely, sprouting caused a significant (*p* < 0.05) decrease of more than 60% in ferulic acid within the bound fraction.

## 4. Discussion

### 4.1. Role of H_2_O_2_ and Germination Time in Promoting Structural Development of White Corn Sprouts

H_2_O_2_ soaking treatment promoted the growth and structural development of white corn sprouts, as evidenced by the significantly greater root and mesocotyl/coleoptile lengths observed in the optimal treatment compared with the germinated control. Escobar-Zuñiga [33] reported a positive effect on root and plumule development in white chickpea sprouts following H_2_O_2_ treatment in a concentration-dependent manner. The authors used concentrations of 5, 17.5, 30, and 35 mM at different germination times, where treatments with higher H_2_O_2_ concentrations produced longer roots and plumules in shorter periods of time. Consistent with our findings, the positive effect of H_2_O_2_ on sprout growth has been reported in several studies [9,34]. This response may be attributed to H_2_O_2_ promoting the energy metabolism required for early sprout growth through interactions between redox status and plant hormones, induction of proteins involved in signaling and plant development, and the mobilization of reserve compounds, including sugars derived from stored starch and other structural components [29,35].

### 4.2. Changes in Proximate Composition of White Corn Induced by Germination and H*_2_*O*_2_* Treatment

Germination significantly modified the proximate composition of white corn, particularly by increasing protein content and reducing lipid levels. Additionally, the optimal H_2_O_2_ treatment produced a lower lipid content than the germinated control. The increase in protein content associated with germination may be attributed to dry matter loss, primarily resulting from carbohydrate oxidation during respiration, together with the activation of enzymes involved in protein synthesis throughout the germination process [2]. Linares-Castañeda, et al. [36] reported similar behavior in chickpea sprouts elicited with H_2_O_2_, with the percentage of protein increasing from 20.96% in the germinated seed to 24.39% in the elicited seed. Furthermore, it is possible that H_2_O_2_ promoted the action of gibberellic acid, which participates significantly in the metabolic pathways associated with protein synthesis. This response can be explained by the role of H_2_O_2_ as a signaling molecule that interacts with various phytohormones and regulates physiological processes in plants [37,38]. Similar trends in lipid content have also been reported in different grains, including blue corn, chickpeas, and chia [2,39,40]. This reduction may be attributed to the mobilization and utilization of stored lipids, particularly triacylglycerols, which are hydrolyzed into fatty acids through lipase activity and subsequently metabolized via β-oxidation to provide energy required for the biochemical and physicochemical changes occurring during germination [41]. Moreover, the greater decrease in lipid content observed in the optimal treatment with H_2_O_2_ may be associated with the generation of reactive oxygen species (ROS) during germination, which has been reported to promote reserve mobilization through oxidative modifications of stored lipids and proteins [12]. This phenomenon may also explain the enhanced development of sprout structures observed under the optimal treatment conditions, as shown in Table 5.

### 4.3. H_2_O_2_-Induced Enhancement of Phenolic Compounds and GABA During White Corn Germination

Sprouting significantly modified the distribution of phenolic compounds in corn by increasing the free phenolic fraction while reducing the bound fraction compared with unsprouted corn. However, the TPC increased compared with the control sprouted in response to H_2_O_2_ treatment. This finding is consistent with previosu reports describing the elicitor effect of H_2_O_2_ ads its role in enhancing phenolic compound accumulation in barley ant lettuce under various stress conditions [42,43]. The increase observed in this study was comparable to that reported by León-López, et al. [8] in chickpea sprouts treated with H_2_O_2_ and by Gawlik-Dziki, et al. [44] in elicited wheat, who also observed a greater accumulation of phenolic compounds compared to the sprouted controls. This finding is significant as the antioxidant capacity of phenolic compounds directly contributes to the overall antioxidant capacity. The increase in phenolic compounds during germination agrees with previous reports for white and blue corn [2,45] and legumes [9,39,46].

The observed rise likely results from de novo synthesis and from enzymatic liberation of phenolic compounds, especially hydroxycinnamates such as ferulic and *p*-coumaric acids that are bound by ester and ether linkages to non-starch polysaccharides in the grain cell walls. Esterases and other cell-wall-degrading enzymes act on these bonds during germination, releasing bound phenolics [2]. Additionally, the activation of phenylalanine ammonia-lyase (PAL), the key enzyme in phenolic biosynthesis, has been reported during seed germination. PAL converts L-phenylalanine to t-cinnamic acid, a precursor of simple phenolics (*p*-coumaric, ferulic, and benzoic acids) and more complex phenols (flavonoids and lignins) [47]. The highest content of phenolic compounds was observed when [H_2_O_2_] = 25 mM and Gt = 96 h were applied, indicating that achieving the maximum FPC is achieved under relatively high [H_2_O_2_] combined with prolonged Gt. The increase in phenolic compounds in sprouts pretreated with H_2_O_2_ as an elicitor has been reported by Delis-Hechavarría, et al. [42] in barley. The authors suggested that these results could be attributed to the induction of PAL gene expression or activity. In that study, the authors found increased PAL activity in sprouts pretreated with 50 mM H_2_O_2_, which was associated with an increase in phenolic compounds. Moreover, it has been demonstrated that this enzyme can be activated by various types of stress in seeds and sprouts [48]. Additionally, certain elicitors may induce cell-wall loosening, leading to the release of phenolic compounds previously bound to the wall and to an increase in FPC in the sprouts [49].

The optimal germination treatment significantly enhanced GABA accumulation in white corn sprouts, producing markedly higher levels than both the ungerminated seeds and the germinated control treatment. The increase in GABA content resulting from the use of H_2_O_2_ elicitation represents a novel finding that, to our knowledge, has not yet been reported for white corn grains. In plants, H_2_O_2_ acts as a central signaling molecule that interacts with Ca^2+^, ABA, and GABA to promote seed germination [50,51]. The synthesis and metabolism of GABA are primarily mediated through the GABA shunt, a metabolic branch of the tricarboxylic acid (TCA) cycle involving three key enzymes: glutamate decarboxylase (GAD), γ-aminobutyric acid aminotransferase (GABA-T), and succinate semialdehyde dehydrogenase (SSADH) [52]. Under oxidative stress, ROS such as H_2_O_2_ induce an influx of intracellular Ca^2+^. This accumulation stimulates cytoplasmic GAD activity by promoting its binding to the Ca^2+^/calmodulin (CaM) complex, thereby accelerating the decarboxylation of L-glutamate (Glu) into GABA [53]. In accordance with this mechanism, our results suggest that H_2_O_2_ exerts a significant positive effect (*p* < 0.05) on GABA accumulation when combined with the germination process. However, the influence of different types of biotic and abiotic stress in combination with germination on GABA accumulation has been reported in several grains, where the response may be either positive or negative depending on the stress conditions applied [54,55]. In addition, Chavarín-Martínez, et al. [56] reported up to a threefold increase in GABA content in germinated blue corn grains elicited with UV-B light (280–320 nm) compared with ungerminated seeds (9.80 mg/100 g GABA), with maximum values of 29.38 mg/100 g GABA and 21.25 mg/100 g GABA in the control treatment (germinated without elicitor). These findings are consistent with the results of the present study, where the use of elicitors promoted higher GABA accumulation. The greater GABA content reported by those authors may be attributed to differences among grain types and, particularly, to the longer germination period used in their study (207 h). Furthermore, Al-Quraan and Al-Omari [57] conducted experiments using lentils germinated in synthetic soil supplemented with H_2_O_2_ at different concentrations (75, 200, 500 µM, and 1 mM), reporting increases in GABA accumulation as the H_2_O_2_ concentration increased. These results are consistent with those obtained in the present study when low and medium concentrations of H_2_O_2_ were applied, resulting in increased GABA content. The effect of peroxide on GABA accumulation in germinated grains has not been extensively studied; however, the increase in GABA observed in these investigations may be associated with the elevated production of reactive oxygen species (ROS) caused by oxidative stress induced by H_2_O_2_ at appropriate concentrations. On the other hand, our results showed that GABA content declined as H_2_O_2_ concentration increased. This finding differs from that reported by Guo, Gong, Luo, Zuo and Shen [53], who suggested that cytosolic GABA is transported into the mitochondrial matrix through specific membrane transporters, where it contributes to the reduction in excess ROS and enhances plant tolerance to oxidative stress. However, Islam, et al. [58] reported that ROS-induced alterations in phytohormone levels are associated with changes in GABA metabolism, suggesting complex interactions among multiple signaling pathways that collectively contribute to plant stress tolerance.

The decrease in GABA content at high [H_2_O_2_] may be attributed to ROS-induced oxidative damage, which can reduce the activity of enzymes involved in GABA biosynthesis. A similar effect was reported by Delis-Hechavarría, et al. [42], who observed reduced phenylalanine ammonia-lyase (PAL) activity during phenolic compounds synthesis at H_2_O_2_ concentrations of 100 and 150 mM. Furthermore, high concentrations of H_2_O_2_ have been shown to cause oxidative damage to essential cellular metabolites, whereas lower concentrations are involved in cellular signaling [59].

These results and trends are consistent with the antioxidant capacity values determined using the ABTS method in the present study. Notably, the AOxC decreased when the [H_2_O_2_] concentration exceeded 25 mM. The AOxC profile followed a pattern similar to that observed for GABA (Figure 3A,B). It has been reported that GABA participates in plant responses to oxidative stress by enhancing the activities of several antioxidant enzymes, including superoxide dismutase (SOD), peroxidase (POD), ascorbate peroxidase (APX), and catalase (CAT), as well as by promoting the metabolism of non-enzymatic antioxidants such as reduced glutathione (GSH), oxidized glutathione (GSSG), ascorbic acid (ASA), and dehydroascorbic acid (DHA). Consequently, GABA may enhance antioxidant activity by mitigating oxidative damage [60]. The increase in AOxC has generally been associated with the rise in phenolic compounds generated during the germination bioprocess [2,39]. Although phenolic compounds are considered major contributors to antioxidant capacity, the weak relationship observed between AOxC evaluated by the ABTS method (Figure 3B) and FPC (Figure 2B) suggests that other antioxidant compounds may also play an important role. In corn, phenolic acids such as ferulic, *p*-coumaric, syringic, and gallic acids are the most abundant phenolic compounds, with ferulic acid contributing substantially to antioxidant activity [20]. However, germination not only affects the phenolic profile but also promotes the accumulation of other bioactive compounds with antioxidant properties, including GABA, vitamins, and antioxidant peptides [54]. In addition, GABA has been reported to stimulate the biosynthesis of phenolics and carotenoids, which may further enhance the antioxidant potential of germinated grains [61]. Therefore, the antioxidant capacity measured by the ABTS and ORAC assays likely results from the combined action of multiple antioxidant compounds, as well as possible synergistic interactions among them, rather than from phenolic compounds alone.

By contrast, the AOxC values obtained by this method showed a greater percentage increase between sprouts from the optimal and control treatments than those obtained with the ABTS method. This difference may be attributed to the distinct reaction mechanisms underlying the two assays. Therefore, the ORAC assay may have detected the antioxidant activity of additional antioxidant compounds synthesized in response to H_2_O_2_-induced stress, particularly those with greater radical-scavenging capacity, which may not be adequately measured by the ABTS technique. These results are consistent with those reported by Domínguez-Arispuro, et al. [39], who observed a greater increase in antioxidant capacity evaluated by the ORAC method (from 5456 to 15,143 µmol TE/100 g, DW) compared with the ABTS method (from 5871 to 14,435 µmol TE/100 g DW) when evaluating the effect of optimal germination conditions on black chickpea relative to unprocessed seeds.

### 4.4. Influence of Germination-H_2_O_2_ Elicitation on Phenolic Acid Profile and Distribution in White Corn

This study demonstrated that germination significantly modifies the phenolic acid profile of maize. Sprouting activates grain metabolism, promoting the degradation of antinutritional factors and storage macronutrients, while simultaneously stimulating the biosynthesis of phenolic compounds associated with potential health benefits. In recent decades, sprouting has emerged as a promising green food biotechnology approach for enhancing the nutritional quality of cereals and legumes, as well as for increasing the production of secondary metabolites with potential applications in nutraceuticals and functional foods. The sprouting process induces a wide range of biotransformations, encompassing both molecular changes and macroscopic structural modifications [62,63]. Phenolic compounds associated with the cell-wall matrix generally exhibit greater antioxidant capacity than those present in the free fraction. In cereals, the insoluble-bound phenolic fraction represents the major phenolic component and is covalently linked to the plant cell-wall matrix through ester and ether bonds with lignin, polysaccharides (e.g., arabinoxylans), and structural proteins. Due to their incorporation within this macromolecular network, these compounds require enzymatic or chemical hydrolysis for their release. This fraction is of particular physiological relevance because it resists digestion in the upper gastrointestinal tract and reaches the colon, where microbial fermentation converts bound phenolics into bioactive metabolites capable of exerting systemic antioxidant and anti-inflammatory effects [63,64,65,66,67,68].

Studies conducted by Karakas, et al. [69] on different cereal kernels, including einkorn, emmer, durum, and bread wheat, demonstrated that *p*-coumaric acid was the most abundant phenolic compound in all sprouted grains, whereas trans-ferulic acid predominated in unsprouted grains. Consistent with the findings of the present study, these authors also reported that ferulic acid was mainly concentrated within the bound phenolic fraction of unsprouted grains (Table 7). In a study conducted by Baranzelli, Somacal, Monteiro, Mello, Rodrigues, Prestes, López-Ruiz, Garrido Frenich, Romero-González and Miranda [31] on sprouted wheat (72 h), the authors reported a smaller decrease (16%) in bound phenolics compared with the findings of the present study. The optimal treatment increased quercetin content in the bound fraction compared with the unsprouted grains and the control sprouts. Mendoza-Sánchez, et al. [70] also observed a greater accumulation of quercetin in sprouts subjected to elicitation with H_2_O_2_ compared with untreated sprouts. However, bioprocesses such as germination markedly alter both the phenolic profile and the distribution of compounds among the analyzed fractions. Interestingly, after 92 h of germination, caffeic and syringic acids were detected exclusively in the free fraction under both control (0 mM, 92 h) and optimized (20 mM H_2_O_2_ and 92 h) conditions, reaching concentrations of 15.11 and 13.56 µg/g DW for caffeic acid, and 11.68 and 6.12 µg/g DW for syringic acid, respectively. Świeca and Baraniak [71] demonstrated that H_2_O_2_ induction in lentil sprouts increased the concentration of free phenolic acids, including caffeic acid, compared with controls without elicitor, indicating that H_2_O_2_ acts as an oxidative signal involved in the regulation of phenolic metabolism. Świeca [72] reported that H_2_O_2_ treatment enhances the de novo synthesis and release of phenolic compounds in sprouted systems, leading to an overall increase in antioxidant-related metabolites. The appearance of caffeic and syringic acids following sprouting is attributed to germination, a physiological bioprocess capable of promoting both the hydrolysis of matrix-bound phytochemicals and the de novo biosynthesis of secondary metabolites [31,63,73,74]. Enzymatic activation during germination promotes the biosynthesis and release of polyphenolic compounds such as caffeic and syringic acids, while the concentrations of other phenolic acids may decrease due to their transformation or continued association with the cell-wall matrix [67,73].

On the other hand, the increase in phenolic acids and flavonoids in the free fraction may be attributed to the biochemical changes that occur during germination. In particular, the enhanced activity of hydrolytic enzymes, including cellulases, amylases, hemicellulases, glucanases, and polyphenol oxidases, promotes the degradation of cell-wall polymers. This enzymatic activity facilitates the release and transformation of phenolic compounds through the degradation of structural polysaccharides, thereby enhancing their bioavailability, although the magnitude of these effects depends on the seed type [31,63,67,75]. The increase in phenolic acids and flavonoids in the free fraction observed in this study is consistent with that reported in other germinated cereals. Chen, et al. [76] reported an increase in the content of both free and bound phenolic compounds during wheat germination, accompanied by enhanced antioxidant activity. Similarly, Kim- MiJeong, et al. [77] observed progressive increases in free phenolic acids, including gallic, caffeic, vanillic, and syringic acids, during 96 h of wheat germination, attributing these changes to the degradation of cell wall components and the activation of biosynthetic pathways related to phenolic metabolism. Likewise, in rice, the content of free phenolics increased between 58 and 87% during germination, and total flavonoids increased by 23.6% [78].

Furthermore, germination activates several secondary metabolic pathways involved in phenolic biosynthesis, including the oxidative pentose phosphate, glycolytic, acetate-malonate, shikimate, and phenylpropanoid pathways. This metabolic stimulation, together with the increased activity of key enzymes such as phenylalanine ammonia-lyase, contributes to the significant enhancement of phenolic content and antioxidant activity in sprouted kernels [63,67,75]. In addition, germination is strongly influenced by environmental factors such as temperature and humidity, which act as abiotic stressors during sprout development. These conditions promote the generation of reactive oxygen species, thereby activating detoxification mechanisms and stimulating the accumulation of phenolic compounds and antioxidant metabolites involved in the regulation of plant metabolism [79,80].

Conversely, the observed reduction (*p* < 0.05) in the total phenolic content of the bound fraction in sprouted kernels is driven by the activation of stored phenolic compounds through the activation of enzymes such as polyphenol oxidase [67,75]. Sprouted cereals have attracted considerable scientific interest due to their health-promoting properties, which are associated with the enhanced bioavailability of essential nutrients and phenolic antioxidants. Previous studies have highlighted their potential role in supporting metabolic functions and reducing the risk of chronic diseases, including obesity and diabetes [63,67].

## 5. Conclusions

The present study demonstrates that H_2_O_2_ pretreatment prior to white corn prior to germination is an effective and sustainable strategy for enhancing sprout quality and promoting growth. Response surface methodology identified 20 mM H_2_O_2_ and 92 h of germination as the optimal conditions for maximizing the accumulation of GABA and bioactive phenolic compounds, including free gallic acid, as well as bound ferulic acid and quercetin. These conditions also significantly improved antioxidant capacity, as evidenced by increased ABTS•^+^ scavenging activity and ORAC values. Collectively, these findings support the use of H_2_O_2_-assisted germination to produce nutritionally enhanced functional corn flours.

## Figures and Tables

**Table 1:** Central composite rotatable design (CCRD) with 13 experiments for X_1_ = hydrogen peroxide concentration ([H_2_O_2_]) and X_2_ = germination time (Gt).

Assay	Germination Treatments ^1^	
	Process Variables ^2^	
	[H_2_O_2_] (mM) (X_1_)	Gt (h) (X_2_)
1	25 (0)	48 (0)
2	25 (0)	48 (0)
3	0 (−1.414)	48 (0)
4	50 (+1.414)	48 (0)
5	7.5 (−1)	82 (+1)
6	42.5(+1)	14 (−1)
7	7.5 (−1)	14 (−1)
8	25 (0)	48 (0)
9	25 (0)	96 (+1.414)
10	25 (0)	48 (0)
11	25 (0)	48 (0)
12	25 (0)	0 (−1.414)
13	42.5 (+1)	82 (+1)

^1^ Central composite design with two factors and five levels; 13 assays. ^2^ [H_2_O_2_]; H_2_O_2_ soaking concentration, Gt; Germination time; values in parentheses correspond to coded levels.

**Table 2:** Sprout length and diameter, and average number of secondary roots in white corn sprouts after soaking in varying hydrogen peroxide concentrations [H_2_O_2_] and germination for different times (Gt).

No. ^1^	[H_2_O_2_]	Gt	GP (%)	Radicle ^2^		
				Length (mm)	Diameter (mm)	# Secondary Roots
12	25	0	0 ^e^	NP	NP	NP
7	7.5	14	24.00 ^d^	0.07 ^f^	0.14 ^b^	NP
6	42.5	14	49.33 ^c^	0.23 ^e^	0.21 ^b^	NP
3	0	48	69.00 ^b^	21.50 ^e^	0.88 ^a^	1.00 ^c^
1^§^	25	48	93.14 ^a^	25.19 ^d^	0.84 ^a^	1.25 ^c^
4	50	48	94.00 ^a^	30.06 ^d^	0.92 ^a^	1.37 ^c^
5	7.5	82	93.00 ^a^	45.17 ^c^	0.93 ^a^	2.40 ^b^
13	42.5	82	93.33 ^a^	55.00 ^b^	0.91 ^a^	2.80 ^ab^
9	25	96	92.00 ^a^	80.00 ^a^	0.93 ^a^	3.40 ^a^

^1^ Treatment number assigned according to the CCRD. ^§^ Central point repeated by 5. ^2^ Fifty seeds were analyzed per treatment. The symbol “#” denotes the number (count) of seeds exhibiting secondary roots. NP; secondary roots were not present. Different lowercase letters within columns indicate significant differences (Tukey, *p* < 0.05).

**Table 3:** Rotatable central composite design for treatment combinations of H_2_O_2_ concentration ([H_2_O_2_]) and germination time (Gt) in white corn sprout production, with observed values for the selected response variables.

Treatment	Process Variables		Response Variables			
	[H_2_O_2_] (mM)	Gt (h)	GP (%)	FPC (mg GAE/100 g)	GABA (mg/100 g)	AOxC (µmol TE/100 g)
1	25	48	94	117.92	12.41	2946.91
2	25	48	90.6	119.40	11.84	2776.71
3	0	48	69	106.20	10.41	2790.63
4	50	48	94	138.96	10.47	2358.35
5	7.5	82	93	182.77	18.44	5284.25
6	42.5	14	49.3	78.43	7.57	1563.23
7	7.5	14	24	72.42	5.81	1598.65
8	25	48	94	117.43	12.74	2722.75
9	25	96	92	257.26	21.56	6654.12
10	25	48	93	117.50	12.28	2940.30
11	25	48	90	126.04	12.49	2886.31
12	25	0	0	64.08	4.69	1672.94
13	42.5	82	93.3	215.74	16.11	4369.51

[H_2_O_2_]; H_2_O_2_ soaking concentration, Gt; Germination time, GP; germination percentage. FPC; Free phenolic content, GABA; γ-aminobutyric acid, AOxC; antioxidant capacity by the ABTS assay, GAE; gallic acid equivalents, TE; Trolox equivalents. Results are expressed on a dry weight basis (DW).

**Table 4:** Parameters of the predicted quadratic polynomial regression model for response variables—germination percentage (GP), free phenolic content (FPC), γ-aminobutyric acid (GABA), and antioxidant capacity (AOxC)—fitted to the experimental data.

Parameter	Germination Percentage (GP)	Free Phenolic Content (FPC)	γ-Aminobutyric Acid (GABA)	Antioxidant Capacity (AOxC) ^1^
	Coded Values	Coded Values	Coded Values	Coded Values
Intercept β_0_	92.34	119.92	12.35	2854.02
Linear				
β_1_, [H_2_O_2_] (X_1_)	7.67 **	10.72 **	−0.059 ^#^	−195.86 **
β_2_, Gt (X_2_)	30.39 **	65.06 **	5.63 **	1690.71 **
Quadratic				
β_11_, [H_2_O_2_] ($X_{1}^{2}$)	−5.15 **	NS	−0.91 **	−181.90 *
β_22_, Gt ($X_{2}^{2}$)	−22.90 **	19.36 **	0.43 **	611.62 **
Interactive				
β_12_, [H_2_O_2_] × Gt (X_1×2_)	−6.31 **	6.80 *	−1.03 **	−221.91 *
*p*-value for model	<0.0001	<0.0001	<0.0001	<0.0001
*p*-value for lack of fit	0.0858 ^NS^	0.2071 ^NS^	0.1490 ^NS^	0.1174 ^NS^
CV (%)	4.02	3.62	3.81	4.75
R^2^	0.9946	0.9952	0.9945	0.9942
R^2^_ajust_	0.9907	0.9928	0.9905	0.9901

^1^ Determined by the ABTS assay. # *p* ≤ 0.10; * *p* ≤ 0.05; ** *p* ≤ 0.01; NS, not significant (*p* > 0.05).

**Table 5:** Comparison of model-predicted and experimental values obtained under optimal conditions.

Model-Predicted Value	Experimental Value	95% Confidence Interval
GP = 93.00%	GP = 94 ± 1.1%	84.20–101.8%
FPC = 231.22 mg GAE/100 g	FPC = 236.94 ± 15.70 mg GAE/100 g	217.81–244.72 mg GAE/100 g
GABA = 20.69 mg/100 g	GABA = 21.63 ± 1.10 mg/100 g	19.35–22.03 mg/100 g
AOxC = 6194.69 µmol TE/100 g	AOxC = 6311.06 ± 273.44 µmol TE/100 g	5765.20–6626.53 µmol TE/100 g

GP, germination percentage; FPC, Free phenolic content; GABA, γ-aminobutyric acid; AOxC, antioxidant capacity by the ABTS assay; GAE, gallic acid equivalent; TE, Trolox equivalent, DW, dry weight.

**Table 6:** Growth performance, chemical composition, phytochemicals, and antioxidant parameters of unprocessed white corn and sprouts obtained under control and optimal germination conditions.

Property	Unsprouted White Corn	Control Sprouted (0 mM, 92 h)	Optimal Sprouted (20 mM, 92 h)
Growth performance			
GP (%)	-	85 ± 1.5 ^b^	94 ± 1.1 ^a^
Radicle length (mm)	-	64.53 ± 17.66 ^b^	74.70 ± 18.89 ^a^
Mesocotyl/Coleoptile length (mm)	-	19.56 ± 5.01 ^b^	25.23 ± 4.63 ^a^
Mean of secondary roots per seed	-	2.57 ± 1.38 ^a^	2.9 ± 1.18 ^a^
Chemical composition (g/100 g, DW)			
Protein	8.83 ± 0.20 ^b^	9.56 ± 0.32 ^a^	9.65 ± 0.27 ^a^
Lipids	5.71 ± 0.14 ^a^	4.17 ± 0.11 ^b^	2.36 ± 0.03 ^c^
Ashes	1.93 ± 0.01 ^a^	1.41 ± 0.05 ^b^	1.33 ± 0.05 ^b^
Total carbohydrates	83.52 ± 0.24 ^c^	84.84 ± 0.30 ^b^	86.65 ± 0.19 ^a^
Phytochemicals			
Free phenolic content (mg GAE/100 g, DW)	79.24 ± 6.27 ^b^	227.41 ± 5.72 ^a^	236.94 ± 15.70 ^a^
Bound phenolic content (mg GAE/100 g, DW)	218.45 ± 3.90 ^b^	305.51 ± 25.50 ^a^	341.44 ± 15.69 ^a^
Total phenolic content (mg GAE/100 g, DW)	297.69 ± 7.28 ^c^	532.92 ± 21.20 ^b^	578.39 ± 5.71 ^a^
GABA (mg/100 g, DW)	5.98 ± 0.95 ^c^	16.14 ± 2.43 ^b^	21.63 ± 1.10 ^a^
Antioxidant capacity (µmol TE/100 g, DW)			
ABTS			
Free extract	1221.00 ± 103.00 ^c^	5854.03 ± 263.00 ^b^	6311.06 ± 273.44 ^a^
Bound extract	4902.00± 180.00 ^c^	6350.79 ± 223.00 ^b^	6935.41 ± 19.00 ^a^
Total	6123.29 ± 200.00 ^c^	12,204.82 ± 388.00 ^b^	13,246.47 ± 243.00 ^a^
ORAC			
Free extract	2605.77 ± 27.4 ^c^	4922.42 ± 3.77 ^b^	6234.05 ± 134.00 ^a^
Free extract	5675.92 ± 335 ^c^	6941.39 ± 336.00 ^b^	9301.74 ± 74.00 ^a^
Total	8281.69 ± 457 ^c^	11,863.81 ± 92.00 ^b^	15,535.79 ± 209.00 ^a^

Means with different superscripts in the same row show significant differences (*p* < 0.05, Tukey’s HSD). mg GAE/100 g DW; mg of Gallic Acid Equivalents per 100 g DW; µmol TE/100 g DW; μmol of Trolox Equivalents per 100 g DW.

**Table 7:** Phenolic compound profile of unsprouted white corn and white corn sprouted under control and optimal conditions.

Phenolic Compound *	Sample Extract					
	Unsprouted White Corn		Control Sprouted (0 mM, 92 h)		Optimal Sprouted (20 mM, 92 h)	
	Free	Bound	Free	Bound	Free	Bound
Caffeic acid	ND	ND	15.11 ± 0.34 ^a^	ND	11.68 ± 0.19 ^b^	ND
Gallic acid	8.93 ± 0.07 ^e^	8.60 ± 0.04 ^e^	38.95 ± 0.39 ^b^	18.77 ± 0.23 ^c^	49.56 ± 0.26 ^a^	16.46 ± 0.39 ^d^
Ferulic acid	13.42 ± 0.29 ^e^	61.22 ± 0.30 ^a^	20.41 ± 0.32 ^c^	20.01 ± 0.39 ^c^	18.36 ± 0.31 ^d^	24.40 ± 0.45 ^b^
*p*-Coumaric acid	2.14 ± 0.05 ^e^	3.82 ± 0.09 ^c^	3.21 ± 0.14 ^d^	9.33 ± 0.25 ^a^	2.62 ± 0.05 ^e^	5.80 ± 0.07 ^b^
Syringic acid	ND	ND	13.56 ± 0.33 ^a^	ND	6.12 ± 0.18 ^b^	ND
Quercetin	2.80 ± 0.19 ^d^	5.69 ± 0.21 ^b^	4.21 ± 0.13 ^c^	6.04 ± 0.23 ^b^	3.04 ± 0.13 ^d^	10.40 ± 0.32 ^a^
Total	27.29 ± 0.59 ^f^	79.34 ± 0.64 ^c^	95.45 ± 1.66 ^a^	54.15 ± 1.1 ^e^	91.38 ± 1.12 ^b^	57.07 ± 1.22 ^d^

Different lowercase superscript letters indicate significant differences across rows (*p* < 0.05, Tukey’s HSD). ND, not detected. * Expressed as µg/g DW.

## Data Availability

The data supporting the findings of this study are available from the corresponding author, Liliana León-López, lili.leon@uas.edu.mx, upon reasonable request.

## Abbreviations

The following abbreviations are used in this manuscript:

ABTS	2,2′-Azino-bis(3-ethylbenzothiazoline-6-sulfonic acid)
AOAC	Association of Official Analytical Chemists
AOxC	Antioxidant Capacity
AAPH	2,2′-Azobis(2-amidinopropane) dihydrochloride
BFC	Bound Phenolic Content
CCRD	Central Composite Rotatable Design
DW	Dry Weight
DAD	Diode Array Detector
FPC	Free Phenolic Content
GABA	γ-Aminobutyric Acid
GAE	Gallic Acid Equivalents
GP	Germination Percentage
Gt	Germination Time
H_2_O_2_	Hydrogen Peroxide
HPLC	High-Performance Liquid Chromatography
NaOH	Sodium Hydroxide
ORAC	Oxygen Radical Absorbance Capacity
PAL	Phenylalanine Ammonia-Lyase
RSM	Response Surface Methodology
TPC	Total Phenolic Content
TE	Trolox Equivalents
UV-Vis	Ultraviolet–Visible Spectroscopy
